# Association of the clinicopathological characteristics and proteinuria remission of pediatric IgAV with nephrotic-range proteinuria: A retrospective cohort study

**DOI:** 10.3389/fped.2022.959212

**Published:** 2022-10-21

**Authors:** Zhijuan Kang, Mai Xun, Zhihui Li, Zuocheng Yang

**Affiliations:** ^1^ Department of Pediatrics, Third Xiangya Hospital of Central South University, Changsha, China; ^2^ Department of Nephrology, Rheumatology and Immunology, Hunan Children's Hospital, Changsha, China

**Keywords:** IgA vasculitis, nephrotic syndrome, proteinuria, children, prognosis

## Abstract

**Background:**

IgA vasculitis (IgAV) combined with nephrotic-range proteinuria is uncommon, and nephrotic-range proteinuria is considered a risk factor for poor prognosis in children with IgAV. There are few clinical studies with large samples.

**Methods:**

Children with IgAV and nephrotic-range proteinuria who were hospitalized at the Department of Nephrology, Rheumatology and Immunology, Hunan Children's Hospital, from March 2008 to January 2020 were retrospectively studied; the patients were aged ≤18 years and were followed up for ≥12 months. We analyzed clinical characteristics, pathological changes, treatment responses, and their relationships in patients with IgAV combined with nephrotic-range proteinuria.

**Results:**

Two hundred seventy-seven children with an average age at onset of IgAV with nephritis (IgAVN) of 8.0 years (interquartile range (IQR), 6.0–10.0) were enrolled; 65.7% were aged 6–10 years. The male-to-female ratio was 1.35:1. All children had both nephrotic-range proteinuria and hematuria, 49 (17.7%) had hypoalbuminemia, and 9 (3.2%) had estimated glomerular filtration rate  < 90 (mL/min/1.73 m^2^). All included children were followed up for at least 1 year. At 3, 6, and 12 months of follow-up, the remission rates of proteinuria in children with IgAV combined with nephrotic-range proteinuria were 27.8%, 62.1%, and 83.0%, respectively, and the remission rates of hematuria were 1.4%, 8.7%, and 35.7%, respectively. In addition, children with age at onset of IgAV with nephrotic-range proteinuria ≥10 years, who were female, who had proteinuria ≥100 mg/kg/24 h, and who had a pathological grade III or above had lower remission rates of hematuria and proteinuria (*P *< 0.05). Multivariate factor analysis was performed by logistic regression and showed age at onset of IgAVN ≥ 10 years and crescents to be risk factors for nonremission of proteinuria at 12 months of follow-up (*P *< 0.05).

**Conclusions:**

Age at onset of IgAVN, sex, proteinuria level, pathological grade, and crescents significantly affect proteinuria remission in children with IgAV combined with nephrotic-range proteinuria.

## Introduction

IgA vasculitis (IgAV), formerly known as Henoch–Schönlein purpura, is the most common systemic vasculitis in children. Patients with IgAV typically present with a palpable rash, arthritis, and gastrointestinal symptoms, with an estimated incidence of renal impairment ranging from 18%–81% (1, 2). Renal involvement is called IgAV with nephritis (IgAVN), which can manifest as hematuria, proteinuria, renal impairment, and sometimes even nephrotic syndrome (NS) (3). In most cases, IgAV is a self-limiting disease, but some cases progress to end-stage renal disease when the kidneys are involved, resulting in a poor prognosis. In general, the long-term prognosis is closely related to the severity of renal damage.

The clinical manifestations of IgAVN are diverse, but the severity of renal impairment is not completely consistent with the severity of clinical manifestations. Therefore, exploring and clarifying factors affecting the prognosis of IgAVN has been a problem to be solved in recent years. It is now widely accepted that proteinuria is an independent risk factor for renal impairment and that the degree of proteinuria (especially proteinuria >1 g/24 h) is not only a marker of renal damage but also an accelerating factor leading to the progression of renal disease, which is closely related to prognosis (4–6). The development of nephrotic-range proteinuria or persistent NS can affect long-term prognosis and increase the risk of progression to end-stage renal disease (7, 8). Thus, patients with IgAV combined with proteinuria, especially those with nephrotic-range proteinuria, need to be monitored closely. As reductions of proteinuria and its duration are goals of clinical treatment and an important indicator of treatment efficacy, exploring the factors associated with proteinuria remission can help clarify the prognosis of patients with this disease.

This study retrospectively analyzed the clinical and pathological characteristics of 277 children with IgAV combined with nephrotic-range proteinuria and preliminarily investigated relationships among clinical characteristics, pathological changes, and remission of hematuria and proteinuria in children.

## Materials and methods

### Study design

Children with clinical manifestations of IgAV combined with nephrotic-range proteinuria at the Department of Nephrology, Rheumatology and Immunology of Hunan Children's Hospital from March 2008 to January 2020 were retrospectively analyzed. Medical histories, laboratory tests, renal pathological findings, and follow-up results were examined, and hematuria and proteinuria remission was evaluated at 3, 6, and 12 months of follow-up.

### Patients

The inclusion criteria for this pediatric study were as follows: (1) a history of IgAV within 6 months; (2) nephrotic-range proteinuria; (3) follow-up time ≥12 months; and (4) age at onset of IgAVN ≤ 18 years. The exclusion criteria were as follows: (1) diagnosed with another primary or secondary nephritis; (2) incomplete data; and (3) combined with genetic mutations.

### Definitions

Nephrotic-range proteinuria was defined as urine protein quantification ≥50 mg/kg/24 h, hypoalbuminemia as a serum albumin level ≤25 g/L, proteinuria remission as negative proteinuria on ≥3 consecutive urine tests, and normal 24-h urine protein quantification (24-h urine protein quantification <150 mg), and hematuria remission as normal red blood cells (<3 red blood cells/HPF) on ≥3 consecutive urine tests. The glomerular filtration rate was estimated using the Schwartz formula: creatinine clearance (mL/min/1.73 m^2^) = K × L/Pcr, where L is body length (cm) and Pcr is plasma creatinine (*μ*mol); K: full term, <1 year, K is 39.8; 2–12 years, K is 48.6; 13–21 years (female), K is 48.6; and 13–21 years (male), K is 61.9.

### Data collection

General information (sex, age, weight, follow-up time), clinical symptoms (joints involvement, gastrointestinal tract involvement), laboratory parameters (routine blood tests, serum albumin levels, serum creatinine levels, blood urea nitrogen levels, immunoglobulin levels, complement levels), and renal pathology and treatment responses (remission rate of hematuria and proteinuria at 3/6/12 months of treatment) were assessed.

### Histopathology

Pathological tissues were evaluated and diagnosed by two pathologists; pathological results based on light microscopy, immunofluorescence, and electron microscopy were collected. Renal biopsies were completed within 1 week of admission, and all specimens were examined by light microscopy, immunofluorescence microscopy, and electron microscopy. Biopsy sections containing eight or more glomeruli were considered sufficient for histological analysis, and the biopsy results were classified according to the renal pathological definitions of the International Study of Kidney Disease in Children (ISKDC) (9) and the 2016 Oxford classification (MEST-C scores) (10).

### Treatment

All included children presented with nephrotic-range proteinuria and were given a uniform treatment regimen: full-dose steroid therapy combined with mycophenolate mofetil (MMF). Steroid therapy (2 mg/kg/d) was started and gradually reduced after 4 weeks of full-dose treatment and discontinued at approximately 6 months. MMF was administered at a dose of 20–30 mg/kg/d. Patients with grade IIIb lesions and severe lesion proliferation (patients with a percentage of crescents ≥25%) received methylprednisolone pulse therapy. As MMF therapy is sometimes ineffective, treatment with other immunosuppressive agents, such as tacrolimus, cyclosporine A (CsA), and cyclophosphamide, or in combination with these immunosuppressive agents may have been provided.

### Statistical analyses

All data were analyzed and processed by SPSS 25.0 statistical software. Measurement data are expressed as the median with interquartile range (IQR), and the Wilcoxon rank sum test was used to compare groups. Enumeration data are expressed as the number of cases and percentage, and the chi-square test was used for comparisons between groups.

Risk factors that may affect prognosis reported in the literatures and our main research results were modeled, and multivariate factor analysis was performed to explore the relationship between clinicopathological characteristics and proteinuria remission by logistic regression analysis. The results are described as odds ratios (ORs) with 95% confidence intervals (95% CIs). *P*<0.05 were considered statistically significant.

## Results

### Baseline characteristics

**Figure 1:**
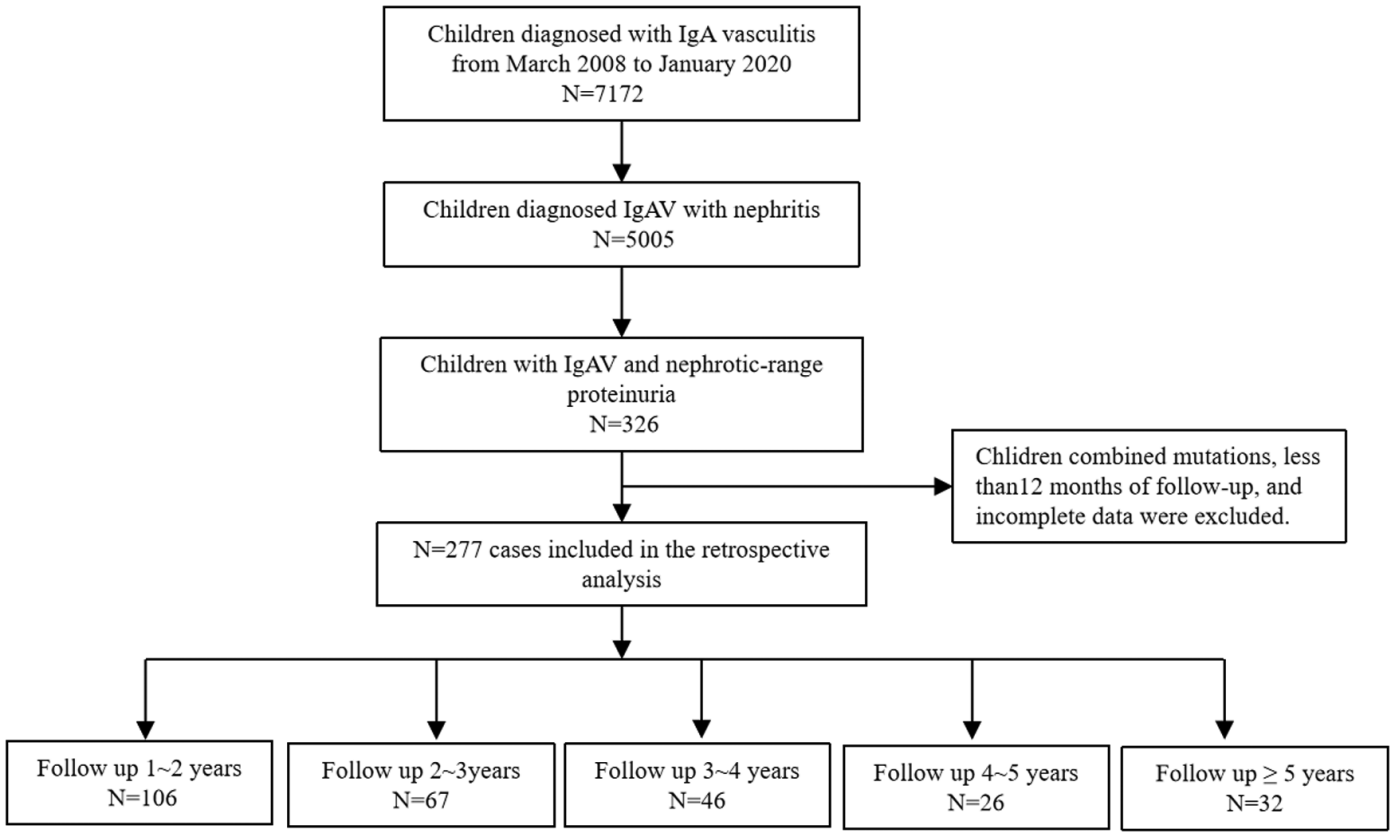
Flow chart for the enrolled patients.

A retrospective analysis of 7,172 children with IgAV admitted to our department from March 2008 to January 2020 was performed. Of these, 5,005 (79.8%) patients were diagnosed with IgAVN, and further analysis identified 326 patients with IgAV complicated by nephrotic-range proteinuria. Those with concurrent genetic mutations and who had less than 12 months of follow-up and incomplete data were excluded. In total, 277 patients were ultimately included in the analysis (Figure 1).

**Figure 2:**
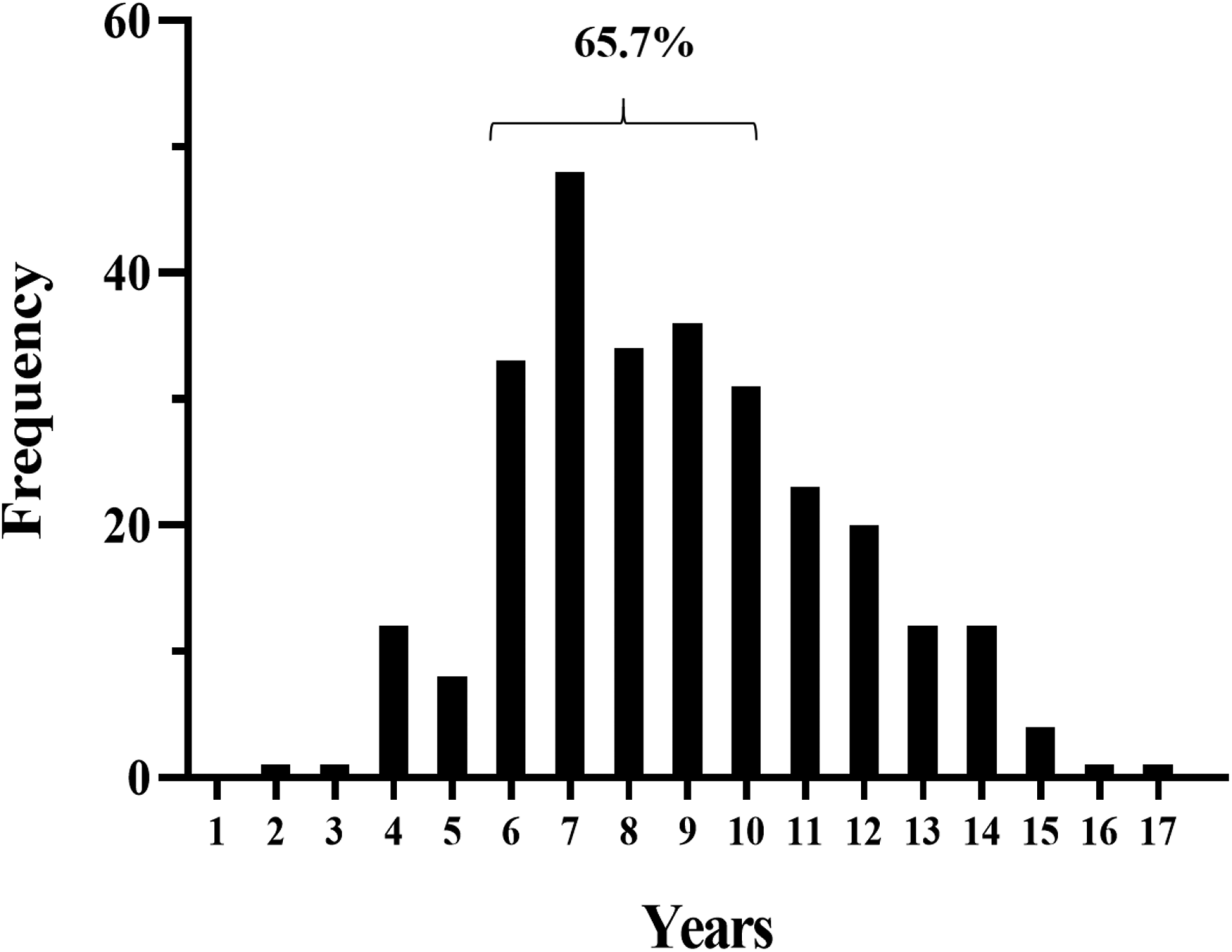
Age distribution of the included children.

The patient characteristics are summarized in Table 1. The median follow-up time was 2.04 years (interquartile range (IQR), 1.06–3.06). Among the patients, 159 (57.4%) were male and 118 (42.6%) female, with a male:female ratio of 1.35:1. The age at onset of IgAVN of the included children was 1–17 years old, with a median age of 8.0 years (IQR, 6.0–10.0), and 65.7% of the children were 6–10 years old (Figure 2).

**Table 1:** Baseline characteristics of the study patients.

Characteristics	All patients (*N* = 277)
Male/female (*n*)	159/118
Age at onset of IgAVN (years)	8.0 (6.0–10.0)
Follow-up time (years)	2.04 (1.06–3.06)
Serum albumin (g/L)	32.18 (27.40–36.35)
Serum albumin ≤25 g/L (%)	49 (17.7%)
eGFR (mL/min/1.73 m^2^)	173.89 (143.40–207.75)
eGFR < 90 (mL/min/1.73 m^2^) (%)	9 (3.2%)
IgA (g/L)	2.03 (1.58–2.55)
Proteinuria (mg/kg/24 h)	84.14 (64.48–113.50)
Hematuria (%)	277 (100%)
Abdominal pain (%)	87 (31.4%)
Arthralgias (%)	30 (10.8%)
ISKDC grade (%)	
IIa	38 (13.7%)
IIb	52 (18.8%)
IIIa	45 (16.2%)
IIIb	137 (49.5%)
IVb	5 (1.8%)
MEST-C score (%)	
M1	67 (24.2%)
E1	147 (53.1%)
S1	50 (18.1%)
T1	2 (0.7%)
C1	149 (53.8%)
C2	38 (13.7%)
Treatment (%)	
Steroid	277 (100%)
Mycophenolate mofetil	277 (100%)
Methylprednisolone pulse	75 (27.1%)
Tacrolimus	73 (26.4%)
Cyclophosphamide	4 (1.4%)
Cyclosporine A	4 (1.4%)

M, mesangial hypercellularity; E, endocapillary proliferation; S, segmental sclerosis/adhesion; T, tubular atrophy/interstitial fibrosis; C, cellular or fibrocellular crescents; IgAVN, IgA vasculitis with nephritis; eGFR, estimated glomerular filtration rate; ISKDC, International Study of Kidney Disease in Children.

All children had both nephrotic-range proteinuria and hematuria. Their median proteinuria level was 84.14 mg/kg/24 h (IQR, 64.48–113.50), median serum albumin level was 32.18 g/L (IQR, 27.40–36.35), and median estimated glomerular filtration rate (eGFR) level was 173.89 mL/min/1.73 m^2^ (IQR, 143.40–207.75). Among the children, 87 (31.4%) had abdominal pain, 30 (10.8%) had arthralgia, and 49 (17.7%) had hypoalbuminemia. Nine (3.2%) patients had eGFR < 90 (mL/min/1.73 m^2^).

Renal biopsies were completed within 1 week of admission, and all included children were evaluated for ISKDC classification; among them, 38 (13.7%) children had grade IIa, 52 (18.8%) grade IIb, 45 (16.2%) grade IIIa, 137 (49.5%) grade IIIb, and 5 (1.8%) grade IVb lesions. MEST-C scores (M: mesangial hypercellularity, E: endocapillary proliferation, S: segmental sclerosis/adhesion, T: tubular atrophy/interstitial fibrosis, C: cellular or fibrocellular crescents) were also used to assess pathological changes, and we found that M1, E1, S1, T1, C1, and C2 occurred in 24.2%, 53.1%, 18.1%, 0.7%, 53.8%, and 13.7%, respectively. All children were treated with steroids + MMF after diagnosis; 75 (27.1%) were treated with additional methylprednisolone pulse because of severe lesion proliferation (patients with a percentage of crescents ≥25%), and 73 (26.4%) were treated with tacrolimus or in combination with tacrolimus because of poor treatment effect. Four (1.4%) children were treated with cyclophosphamide or CsA.

### Follow-up

The children were followed up for a total of 1–9 years, including 106 children with 1–2 years, 67 with 2–3 years, 46 with 3–4 years, 26 with 4–5 years, and 32 with more than 5 years (Figure 1). We uniformly evaluated remission of hematuria and proteinuria after 3, 6, and 12 months of treatment.

**Figure 3:**
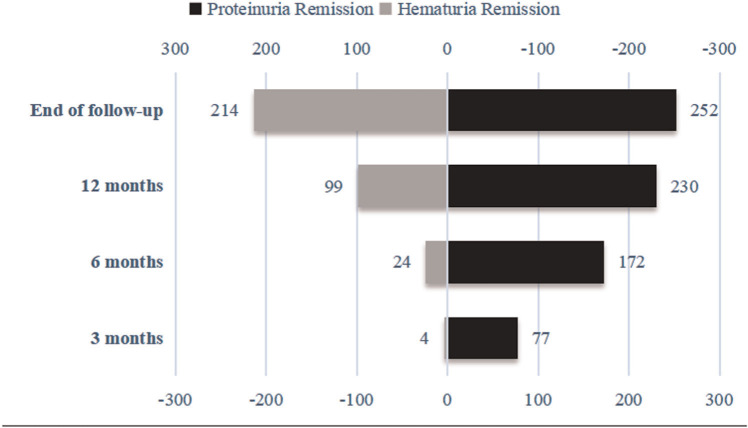
Remission of hematuria and proteinuria.

After 3 months of follow-up, 77 (27.8%) of the 277 children with IgAVN showed complete remission of proteinuria, and 4 (1.4%) experienced hematuria remission. After 6 months of follow-up, 172 (62.1%) children with IgAVN had complete remission of proteinuria, and 24 (8.7%) had remission of hematuria. At 12 months of follow-up, 230 (83.0%) children with IgAVN were in complete remission of proteinuria; 99 (35.7%) showed hematuria remission. At the end of the follow-up, 252 (91.0%) children had complete remission of proteinuria, and hematuria was alleviated in 214 (77.3%) (Figure 3).

### Influences of onset age on the severity of IgAV combined with nephrotic-range proteinuria

To explore the effect of age at onset on IgAVN, we compared children younger than 10 years of age with those older than 10 years of age (Table 2). We found that of the 277 children, 76 (27.4%) and 201 (72.6%) had age at onset ≥10 and <10 years, respectively. Comparing the two groups, children with age at onset of IgAVN ≥10 years had lower rates of proteinuria remission at the 3-, 6-, and 12-month follow-ups; however, there was a significant difference in the rate of hematuria remission only at the 3-month follow-up (*P *< 0.05). There were no significant differences in the clinical characteristics of the two groups (*P > *0.05). Analysis of pathological changes revealed that children with age at onset of IgAVN ≥10 years had a greater proportion of extensive fusion of the foot process and a lower proportion of M1 (*P *< 0.05). In terms of treatment, children with age at onset of IgAVN ≥10 years were more likely to receive tacrolimus (*P *< 0.05).

**Table 2:** Effects of onset age and sex on the severity of IgAV combined with nephrotic-range proteinuria.

	Age at onset of IgAVN		*P*-value	Sex		*P-*value
	<10 years	≥10 years		Female	Male	
	*N* = 201	*N* = 76		*N* = 118	*N* = 159	
Clinical characteristics						
Male/female (n)	118/83	41/35	0.475	—	—	
Age at onset of IgAVN (years)	—	—		8.01 (6.03–10.04)	8.01 (6.05–10.00)	0.973
Follow-up time (years)	2.03 (1.06–3.06)	2.05 (1.08–3.04)	0.690	2.05 (1.06–3.07)	2.02 (1.06–3.03)	0.280
eGFR (mL/min/1.73 m^2^)	173.89 (145.15–205.74)	175.20 (141.86–208.20)	0.983	172.57 (138.02–209.14)	176.18 (144.71–206.67)	0.694
Serum albumin (g/L)	32.20 (27.70–36.20)	31.60 (26.47–36.80)	0.773	32.16 (25.30–36.30)	32.20 (28.60–36.40)	0.282
Serum albumin ≤25 g/L (%)	34 (16.9%)	15 (19.7%)	0.583	27 (22.9%)	22 (13.8%)	0.051
IgA (g/L)	1.97 (1.59–2.46)	2.27 (1.53–2.82)	0.139	2.00 (1.53–2.46)	2.04 (1.63–2.65)	0.299
Abdominal pain (%)	67 (33.3%)	20 (26.3%)	0.262	30 (25.4%)	57 (35.8%)	0.065
Arthralgias (%)	24 (11.9%)	6 (7.9%)	0.334	10 (8.5%)	20 (12.6%)	0.277
Proteinuria (mg/kg/24 h)	82.93 (64.26–113.51)	85.48 (65.27–111.43)	0.817	86.64 (66.18–113.49)	81.68 (62.29–113.42)	0.335
Pathological changes (%)						
Foot process lesions	198 (98.5%)	74 (97.4%)	0.525	116 (98.3%)	156 (98.1%)	0.906
Segmental fusion of foot process	155 (77.1%)	49 (64.5%)	0.033	86 (72.9%)	118 (74.2%)	0.803
Extensive fusion of foot process	42 (20.9%)	25 (32.9%)	0.037	29 (24.6%)	38 (23.9%)	0.896
Pathological grade			0.626			0.100
Grade II	67 (33.3%)	23 (30.3%)		32 (27.1%)	58 (36.5%)	
Grade III and above	134 (66.7%)	53 (69.7%)		86 (72.9%)	101 (63.5%)	
MEST-C score						
M1	57 (28.4%)	10 (13.2%)	0.008	32 (27.1%)	35 (22.0%)	0.326
E1	107 (53.2%)	40 (52.6%)	0.929	60 (50.8%)	87 (54.7%)	0.523
S1	40 (19.9%)	10 (13.2%)	0.193	24 (20.3%)	26 (16.4)	0.394
C1 and C2	134 (66.7%)	53 (69.7%)	0.626	86 (72.9%)	101 (63.5%)	0.100
Treatment (%)						
Methylprednisolone pulse	56 (27.9%)	19 (25.0%)	0.633	38 (32.2%)	37 (23.3%)	0.098
Tacrolimus	45 (22.4%)	28 (36.8%)	0.015	41 (34.7%)	32 (20.1%)	0.006
Follow-up (%)						
Follow-up for 3 months						
Proteinuria remission	66 (32.8%)	11 (14.5%)	0.002	24 (20.3%)	53 (33.3%)	0.017
Hematuria remission	1 (0.5%)	3 (3.9%)	0.032	1 (0.8%)	3 (1.9%)	0.473
Follow-up for 6 months						
Proteinuria remission	136 (67.7%)	36 (47.4%)	0.002	66 (55.9%)	106 (66.7%)	0.069
Hematuria remission	16 (8.0%)	8 (10.5%)	0.498	8 (6.8%)	16 (10.1%)	0.337
Follow-up for 12 months						
Proteinuria remission	176 (87.6%)	54 (71.1%)	0.001	94 (79.7%)	136 (85.5%)	0.198
Hematuria remission	74 (36.8%)	25 (32.9%)	0.543	34 (28.8%)	65 (40.9%)	0.038

M, mesangial hypercellularity; E, endocapillary proliferation; S, segmental sclerosis/adhesion; T, tubular atrophy/interstitial fibrosis; C, cellular or fibrocellular crescents; IgAV, IgA vasculitis; IgAVN, IgA vasculitis with nephritis; eGFR, estimated glomerular filtration rate.

### Influences of sex on the severity of IgAV combined with nephrotic-range proteinuria

The patients were divided into two groups according to sex, 159 males and 118 females, and clinical characteristics, pathological changes, and treatment responses were compared (Table 2). Males had a higher rate of proteinuria remission at the 3-month follow-up than females (*P *< 0.05), but there was no significant difference in the rate of proteinuria remission between the two groups at the 6- and 12-month follow-ups. Unlike for proteinuria remission, the rate of hematuria remission at the 3- and 6-month follow-ups in males was not significantly different from that in females, though the rate of hematuria remission at 12 months was higher in males (*P *< 0.05). There were no significant differences in clinical characteristics or pathological changes between the two groups (*P > *0.05). In terms of treatment, females were more likely to receive tacrolimus than males (*P *< 0.05).

### Influences of the degree of proteinuria on the severity of IgAV combined with nephrotic-range proteinuria

All 277 children who presented with nephrotic-range proteinuria were further divided into two groups according to the degree of proteinuria: proteinuria <100 mg/kg/24 h and proteinuria ≥100 mg/kg/24 h (180/277, 65.0% vs. 97/277, 35%) groups (Table 3). Compared to those in the proteinuria <100 mg/kg/24 h group, the patients in the proteinuria ≥100 mg/kg/24 h group had lower serum albumin levels, a higher incidence of hypoalbuminemia, and a higher proportion of M1 among pathological changes and were more likely to receive methylprednisolone pulse and tacrolimus (*P *< 0.05). There were no significant differences in other clinical characteristics and pathological changes. Analysis of treatment responses showed that the proteinuria ≥100 mg/kg/24 h group had a lower rate of proteinuria remission at the 3- and 6-month follow-ups. Nonetheless, only the rate of hematuria remission at 12 months differed between the two groups, with the rate being significantly lower in the proteinuria ≥100 mg/kg/24 h group (*P *< 0.05).

**Table 3:** Effects of proteinuria and pathological grade on the severity of IgAV combined with nephrotic-range proteinuria.

	Proteinuria		*P*-value	Pathological grade		*P*-value
	<100 mg/kg/24 h	≥100 mg/kg/24 h		Grade II	Grade III and above	
	*N* = 180	*N* = 97		*N* = 90	*N* = 187	
Clinical characteristics						
Male/female (n)	108/72	51/46	0.233	58/32	101/86	0.100
Age at onset of IgAVN (years)	8.02 (6.05–10.01)	7.11 (6.03–10.04)	0.544	7.09 (6.02–10.00)	8.03 (6.05–10.05)	0.301
Follow-up time (years)	2.04 (1.06–3.07)	2.04 (1.06–3.05)	0.735	2.03 (1.05–3.06)	2.04 (1.07–3.06)	0.463
eGFR (mL/min/1.73 m^2^)	181.85 (152.07–210.53)	162.56 (135.63–193.83)	0.006	191.48 (162.97–209.42)	166.18 (139.71–199.69)	0.004
Serum albumin (g/L)	33.60 (30.20–37.20)	28.50 (23.95–33.70)	0.000	34.20 (29.70–37.64)	31.50 (26.47–35.64)	0.004
Serum albumin ≤ 25 g/L (%)	20 (11.1%)	29 (29.9%)	0.000	11 (12.2%)	38 (20.3%)	0.098
IgA (g/L)	2.07 (1.63–2.70)	1.96 (1.50–2.40)	0.166	2.19 (1.53–2.62)	1.99 (1.60–2.48)	0.585
Abdominal pain (%)	55 (30.6%)	32 (33.0%)	0.677	24 (26.7%)	63 (33.7%)	0.238
Arthralgias (%)	20 (11.1%)	10 (10.3%)	0.838	12 (13.3%)	18 (9.6%)	0.352
Proteinuria (mg/kg/24 h)	—	—	—	79.89 (61.88–109.84)	85.62 (65.95–113.87)	0.250
Pathological changes (%)						
Foot process lesions	178 (98.9%)	94 (96.9%)	0.237	88 (97.8%)	184 (98.4%)	1.000
Segmental fusion of foot process	137 (76.1%)	67 (69.1%)	0.205	76 (84.4%)	128 (68.4%)	0.005
Extensive fusion of foot process	40 (22.2%)	27 (27.8%)	0.298	12 (13.3%)	55 (29.4%)	0.003
Pathological grade			0.499			—
Grade II	61 (33.9%)	29 (29.9%)		—	—	—
Grade III and above	119 (66.1%)	68 (70.1%)		—	—	—
MEST-C score						
M1	35 (19.4%)	32 (33.0%)	0.012	15 (16.7%)	52 (27.8%)	0.043
E1	89 (49.4%)	58 (59.8%)	0.100	46 (51.1%)	101 (54.0%)	0.651
S1	35 (19.4%)	15 (15.5%)	0.411	7 (7.8%)	43 (23.0%)	0.002
C1 and C2	119 (66.1%)	68 (70.1%)	0.499	0 (0.0%)	187 (100.0%)	0.000
Treatment (%)						
Methylprednisolone pulse	36 (20.0%)	39 (40.2%)	0.000	0 (0.0%)	75 (40.1%)	0.000
Tacrolimus	39 (21.7%)	34 (35.1%)	0.016	22 (24.4%)	51 (27.3%)	0.617
Follow-up (%)						
Follow-up for 3 months						
Proteinuria remission	60 (33.3%)	17 (17.5%)	0.005	33 (36.7%)	44 (23.5)	0.022
Hematuria remission	2 (1.1%)	2 (2.1%)	0.527	2 (2.2%)	2 (1.1%)	0.829
Follow-up for 6 months						
Proteinuria remission	121 (67.2%)	51 (52.6%)	0.017	60 (66.7%)	112 (59.9%)	0.276
Hematuria remission	19 (10.6%)	5 (5.2%)	0.127	9 (10.0%)	15 (8.0%)	0.584
Follow-up for 12 months						
Proteinuria remission	153 (85.0%)	77 (79.4%)	0.235	82 (91.1%)	148 (79.1%)	0.013
Hematuria remission	73 (40.6%)	26 (26.8%)	0.023	45 (50%)	54 (28.9%)	0.001

M, mesangial hypercellularity; E, endocapillary proliferation; S, segmental sclerosis/adhesion; T, tubular atrophy/interstitial fibrosis; C, cellular or fibrocellular crescents; IgAV, IgA vasculitis; IgAVN, IgA vasculitis with nephritis; eGFR, estimated glomerular filtration rate.

### Influences of pathological grade on the severity of IgAV combined with nephrotic-range proteinuria

To further evaluate the influence of pathological grade on IgAVN patients, the patients were divided into two groups: grade II and grade III and above (Table 3). Of the 277 children, 90 (32.5%) were included in the grade II group and 187 (67.5%) in grade III and above group. Comparing clinical characteristics, other pathological damage, and treatment responses between the two groups, we found that patients in grade III and above group had lower serum albumin levels (*P *< 0.05). With regard to pathological lesions, the grade III and above group had a higher proportion of extensive fusion of the foot process, a lower proportion of segmental fusion of the foot process, and a higher proportion of M1, S1, and C1 and C2 (*P *< 0.05). Evaluation of treatment responses revealed lower remission rates of proteinuria and hematuria in grade III and above group. However, the remission rate of proteinuria only differed at the 3- and 12-month follow-ups, and the remission rate of hematuria was only statistically significant between the two groups at the 12-month follow-up. Moreover, children who received methylprednisolone pulse were all classified into the grade III and above group.

### Multivariate analysis of risk factors for nonremission of proteinuria at 3, 6, and 12 months of follow-up

Age at onset of IgAVN, sex, serum albumin level, pathological grade, MEST-C scores, an extensive fusion of the foot processes, and degree of proteinuria were included to construct a multivariate logistic regression equation. The results showed that at 3 months of follow-up, age of IgAVN onset ≥10 years, female sex, serum albumin level <25 g/L, and M1 were risk factors for nonremission of proteinuria. With longer follow-up, only age at onset of IgAVN ≥ 10 years, serum albumin level <25 g/L, high degree of proteinuria, and crescents were risk factors for nonremission of proteinuria at 6 months. At 12 months of follow-up, age at onset of IgAVN ≥ 10 years and crescents were risk factors for nonremission of proteinuria in regression analysis (Table 4).

**Table 4:** Multivariate risk factor analysis for nonremission of proteinuria.

Variables	3 months of follow-up	*P*-value	6 months of follow-up	*P-*value	12 months of follow-up	*P*-value
	OR (95% CI)		OR (95% CI)		OR (95% CI)	
Age at onset of IgAVN (<10 years = 0, ≥10 years = 1)	2.660 (1.262–5.604)	0.010	2.220 (1.229–4.010)	0.008	2.535 (1.244–5.166)	0.010
Sex (male = 0, female = 1)	1.881 (1.032–3.429)	0.039	1.353 (0.788–2.322)	0.273	1.157 (0.578–2.318)	0.680
Serum albumin (≥25 g/L = 0, <25 g/L = 1)	3.576 (1.239–10.324)	0.018	3.054 (1.442–6.468)	0.004	1.664 (0.693–3.994)	0.254
Pathological grades (Grade IIa = 1, IIb = 2, IIIa = 3, IIIb = 4, IVb = 5)	1.365 (0.853–2.186)	0.195	0.693 (0.449–1.068)	0.097	0.750 (0.438–1.286)	0.296
Extensive fusion of foot process (no = 0, yes = 1)	1.302 (0.609–2.784)	0.496	1.027 (0.531–1.985)	0.938	1.174 (0.533–2.587)	0.690
Proteinuria (50–100 mg/kg/24 h = 1,100–150 mg/kg/24 h = 2, 150–200 mg/kg/24 h = 3,200–250 mg/kg/24 h = 4, 250 mg/kg/24 h– = 5)	1.285 (0.878–1.882)	0.197	1.449 (1.056–1.988)	0.022	1.234 (0.846–1.802)	0.275
C (C0 = 0, C1 = 1, C2 = 2)	1.129 (0.487–2.619)	0.777	2.789 (1.323–5.880)	0.007	3.392 (1.477–7.789)	0.004
M (M0 = 0, M1 = 1)	0.426 (0.210–0.861)	0.018	0.885 (0.453–1.730)	0.721	0.717 (0.305–1.690)	0.447
E (E0 = 0, E1 = 1)	1.246 (0.697–2.228)	0.457	1.077 (0.627–1.850)	0.787	1.276 (0.638–2.552)	0.491
S (S0 = 0, S1 = 1)	0.572 (0.273–1.197)	0.138	0.534 (0.253–1.128)	0.100	1.077 (0.436–2.662)	0.872

M, mesangial hypercellularity; E, endocapillary proliferation; S, segmental sclerosis/adhesion; C, cellular or fibrocellular crescents; IgAVN, IgA vasculitis with nephritis; OR, odds ratio; CI, confidence interval.

## Discussion

IgAVN is the most common secondary glomerulonephritis in children, and approximately 1% of these patients develop the end-stage renal disease (11). Several studies have shown that nephrotic-range proteinuria or an NS state at disease onset may be associated with a poor prognosis (2, 8, 12, 13). Therefore, children with IgAV combined with nephrotic-range proteinuria or presenting with NS need to be closely monitored, but there are still relatively few studies involving large samples of such children.

In the present study, we retrospectively analyzed 277 children with IgAV combined with nephrotic-range proteinuria. Although proteinuria remission is not a substitute for renal function, it is still used clinically as an important indicator of treatment effects or even clinical cure. Therefore, remission of proteinuria and hematuria was used to evaluate treatment effects in the children in this study. After 3 months of follow-up, remission rates of proteinuria and hematuria were 27.8% and 1.4%, respectively; after 12 months of follow-up, the rates reached 83.0% and 35.7%, respectively. This result is better than that of previous reports (2, 13), probably due to the earlier renal biopsy and more aggressive treatment of the children in the present study. In a previous study of IgAVN, Park, Tanaka, and Shin JI suggested that early and adequate steroid and/or immunosuppressive therapy may be effective at relieving the symptoms and improving the prognosis of these children. However, due to the retrospective nature of our study and the short follow-up period, no definite conclusions can be drawn (14–16).

Age at onset of IgAVN was previously reported to be 4–6 years (17), whereas 65.7% of the children in this study had an age of onset of 6–10 years, which is higher than previous reports and suggests that onset age may be related to IgAVN prognosis. Therefore, we further divided the included children into two groups according to age at onset of IgAVN: <10 and ≥10 years. Comparative analysis revealed no significant difference in clinical characteristics between these groups, but when assessing pathological changes, an extensive fusion of the foot process was more common and M1 less common in the group age at onset of IgAVN ≥ 10 years. In addition, remission rates of hematuria and proteinuria were significantly lower in children in the age at onset of IgAVN ≥ 10 years group. Studies on the effect of age on the clinical presentation and prognosis of IgAVN have been previously reported. For example, Hennies et al. (18) analyzed data for 202 children with IgAVN registered at 26 centers in the German Society of Pediatric Nephrology from 2008 to 2014 and found that clinical presentation was age-related and that children over 10 years of age had a more impaired renal function and chronic histopathological lesions. Liao et al. (19) retrospectively studied the records of 484 patients under 18 years old who were diagnosed with IgAV in a tertiary care center in Taiwan from January 1999 to December 2018, with age at onset grouped as ≤6 years, 6–12 years (>6 years, ≤12 years), and 12–18 years (>12 years, <18 years). The authors found that pediatric IgAV at different ages of onset had different clinical presentations and prognoses, with the risk of steroid dependence, refractory disease, and renal involvement at disease onset increasing with age.

Of the 277 children in this study, 118 (42.6%) were female. Previous studies have found that the female sex is a risk factor for poor prognosis of IgAVN (2, 20). In the present study, there were no significant differences in clinical manifestations (abdominal pain, arthralgia, rate of hypoalbuminemia, degree of proteinuria, etc.), histopathological changes, or the rate of hematuria remission in the female group compared to the male group, but the early proteinuria remission rate of females was significantly lower than that of males (20.3% vs*.* 33.3%). Nevertheless, there was no statistically significant difference in proteinuria remission rates between the two groups at the 6- and 12-month follow-ups, which is inconsistent with previous studies and may also be because the present study focused on children with IgAV combined with nephrotic-range proteinuria rather than the entire population of children with IgAVN. Regardless, further study of the effect of sex on IgAVN disease is warranted.

The impact of pathological changes on the prognosis of IgAVN has been the focus of clinical research and an important reference indicator for clinical treatment. Clavé et al. (21) studied 170 children with biopsy-proven IgAVN diagnosed between 2007 and 2017, with patients with NS at presentation and those with cellular crescents and chronic lesions at initial renal biopsy being more likely to develop renal damage, highlighting the relationship between clinical and histological manifestations of IgAVN and factors affecting renal regression. In a retrospective study published in 2021, Wang et al. discussed the predictive value of ISKDC classification and MEST-C scores on the prognosis of 877 children with IgAVN and found that S and T could be used to predict renal prognosis (22). However, the impact of renal pathological alterations on IgAVN remains controversial. Xia et al. (23) reviewed the clinical features and prognosis of 101 children with IgAVN with ISKDC classification grades of IIIa/IIIb from January 1992 to November 2008 and found no significant differences in clinical presentation and long-term prognosis between patients with grade IIIa and grade IIIb IgAVN. In this study, the ISKDC standard classified 90 (32.5%) patients as having grade II and 187 (67.5%) as having grade III and above, which indicates that the pathological damage occurring in children with IgAV combined with nephrotic-range proteinuria is serious and that renal biopsy should be completed as soon as possible. According to the Oxford classification, the pathological changes in the children in this study were mainly acute lesions, which may partially explain the children's better response to treatment than in the past. In addition, the present study found that the damage to podocytes differed among different age and pathological groups, emphasizing that podocytes may have an important role in the pathogenesis of IgAVN. In fact, some studies have suggested that IgA nephropathy (IgAN) and IgAV have the same pathogenesis and that the occurrence of IgAN is closely related to podocyte damage (24, 25). The extensive fusion of foot processes found in the children in this study also reminds us that foot processes play a key role in the development of IgAVN and may be a direction for future research.

It is well known that the severity of proteinuria is a prognostic risk factor. In this study, we divided patients into two groups (proteinuria <100 and ≥100 mg/kg/24 h) to investigate the effect of proteinuria on IgAVN and found a significant difference in terms of clinical manifestations and pathological damage, with the children with proteinuria ≥100 mg/kg/24 h having lower serum albumin levels. Interestingly, the degree of proteinuria had little effect on the pathological grading of the patients, but children with high proteinuria accounted for a greater proportion of M1. Further multivariate analysis showed that age at onset of IgAVN ≥10 years, female sex, serum albumin level <25 g/L, and M1 were risk factors for unremitting proteinuria at the 3-month follow-up. At 6 months of follow-up, age at onset of IgAVN ≥10 years, serum albumin level <25 g/L, a high degree of proteinuria, and crescents were risk factors for unremitting proteinuria. In contrast, age at onset of IgAVN ≥10 years and crescents were risk factors for nonremission of proteinuria at 12 months of follow-up. Hence, we may need to focus on different risk factors in different stages of IgAVN. Unfortunately, the effect of treatment on prognosis was ultimately not included in the regression analysis. The reason is that other immunosuppressive agents (tacrolimus, CsA, etc.) were added to this study when steroid + MMF treatment was ineffective, and the addition of these immunosuppressive agents already indicated poor treatment effect. Furthermore, the duration of treatment with other immunosuppressive agents varied greatly among children and was not eligible for inclusion in the analysis.

### Limitations

The present study has some limitations. First, this was only a single-center study with an insufficient follow-up time and lacked a multicenter long-term follow-up. Second, due to the limitations of retrospective studies, hematuria and proteinuria remission rates were selected as evaluation indices of treatment effects. This study lacked a comprehensive evaluation of renal function, which is another important shortcoming.

## Data Availability

The original contributions presented in the study are included in the article/Supplementary Material, further inquiries can be directed to the corresponding authors.

## References

[1] Saulsbury FT. Clinical update: Henoch-Schönlein purpura. Lancet. (2007) 369(9566):976–8. 10.1016/S0140-6736(07)60474-7 17382810

[2] Narchi H. Risk of long term renal impairment and duration of follow up recommended for Henoch-Schonlein purpura with normal or minimal urinary findings: a systematic review. Arch Dis Child. (2005) 90(9):916–20. 10.1136/adc.2005.074641 15871983 PMC1720564

[3] Du L Wang P Liu C Li S Yue S Yang Y. Multisystemic manifestations of IgA vasculitis. Clin Rheumatol. (2021) 40(1):43–52. 10.1007/s10067-020-05166-5 32557258

[4] Taal MW Brenner BM. Renal risk scores: progress and prospects. Kidney Int. (2008) 73(11):1216–9. 10.1038/ki.2008.36 18322541

[5] Verhave JC Gansevoort RT Hillege HL Bakker SJ De Zeeuw D de Jong PE An elevated urinary albumin excretion predicts de novo development of renal function impairment in the general population. Kidney Int Suppl. (2004) 92:S18–21. 10.1111/j.1523-1755.2004.09205.x 15485409

[6] Iseki K Ikemiya Y Iseki C Takishita S. Proteinuria and the risk of developing end-stage renal disease. Kidney Int. (2003) 63(4):1468–74. 10.1046/j.1523-1755.2003.00868.x 12631363

[7] Ronkainen J Ala-Houhala M Huttunen NP Jahnukainen T Koskimies O Ormälä T Outcome of Henoch-Schoenlein nephritis with nephrotic-range proteinuria. Clin Nephrol. (2003) 60(2):80–4. 10.5414/cnp60080 12940608

[8] Wakaki H Ishikura K Hataya H Hamasaki Y Sakai T Yata N Henoch-Schönlein purpura nephritis with nephrotic state in children: predictors of poor outcomes. Pediatr Nephrol. (2011) 26(6):921–5. 10.1007/s00467-011-1827-8 21373776

[9] Counahan R Winterborn MH White RH Heaton JM Meadow SR Bluett NH Prognosis of Henoch-Schönlein nephritis in children. Br Med J. (1977) 2(6078):11–4. 10.1136/bmj.2.6078.11 871734 PMC1631306

[10] Trimarchi H Barratt J Cattran DC Cook HT Coppo R Haas M Oxford classification of IgA nephropathy 2016: an update from the IgA Nephropathy Classification Working Group. Kidney Int. (2017) 91(5):1014–21. 10.1016/j.kint.2017.02.003 28341274

[11] Stewart M Savage JM Bell B McCord B. Long term renal prognosis of Henoch-Schönlein purpura in an unselected childhood population. Eur J Pediatr. (1988) 147(2):113–5. 10.1007/BF00442205 3366130

[12] Coppo R Andrulli S Amore A Gianoglio B Conti G Peruzzi L Predictors of outcome in Henoch-Schönlein nephritis in children and adults. Am J Kidney Dis. (2006) 47(6):993–1003. 10.1053/j.ajkd.2006.02.178 16731294

[13] Goldstein AR White RH Akuse R Chantler C. Long-term follow-up of childhood Henoch-Schönlein nephritis. Lancet. (1992) 339(8788):280–2. 10.1016/0140-6736(92)91341-5 1346291

[14] Park JM Won SC Shin JI Yim H Pai KS. Cyclosporin A therapy for Henoch-Schönlein nephritis with nephrotic-range proteinuria. Pediatr Nephrol. (2011) 26(3):411–7. 10.1007/s00467-010-1723-7 21184240

[15] Tanaka H Suzuki K Nakahata T Ito E Waga S. Early treatment with oral immunosuppressants in severe proteinuric purpura nephritis. Pediatr Nephrol. (2003) 18(4):347–50. 10.1007/s00467-003-1094-4 12700960

[16] Shin JI Park JM Shin YH Kim JH Lee JS Jeong HJ. Henoch-Schönlein purpura nephritis with nephrotic-range proteinuria: histological regression possibly associated with cyclosporin A and steroid treatment. Scand J Rheumatol. (2005) 34(5):392–5. 10.1080/03009740510026544 16234188

[17] Gardner-Medwin JM Dolezalova P Cummins C Southwood TR. Incidence of Henoch-Schönlein purpura, Kawasaki disease, and rare vasculitides in children of different ethnic origins. Lancet. (2002) 360(9341):1197–202. 10.1016/S0140-6736(02)11279-7 12401245

[18] Hennies I Gimpel C Gellermann J Möller K Mayer B Dittrich K Presentation of pediatric Henoch-Schönlein purpura nephritis changes with age and renal histology depends on biopsy timing. Pediatr Nephrol. (2018) 33(2):277–86. 10.1007/s00467-017-3794-1 28983704

[19] Liao CH Tsai M Yang YH Chiang BL Wang LC. Onset age is a risk factor for refractory pediatric IgA vasculitis: a retrospective cohort study. Pediatr Rheumatol Online J. (2020) 18(1):86. 10.1186/s12969-020-00480-3 33172497 PMC7654143

[20] Kiliç BD Demir BK. Determination of risk factors in children diagnosed with Henoch-Schönlein purpura. Arch Rheumatol. (2018) 33(4):395–401. 10.5606/ArchRheumatol.2018.6562 30874233 PMC6409163

[21] Clavé S Sordet M Tsimaratos M Decramer S Fila M Guigonis V Association of kidney biopsy findings with short- and medium-term outcomes in children with moderate-to-severe IgA vasculitis nephritis. Eur J Pediatr. (2021) 180(10):3209–18. 10.1007/s00431-021-04065-4 33934234

[22] Wang M Wang R He X Zhang P Kuang Q Yao J Using MEST-C scores and the international study of kidney disease in children classification to predict outcomes of Henoch-Schönlein purpura nephritis in children. Front Pediatr. (2021) 9:658845. 10.3389/fped.2021.65884 33937154 PMC8079736

[23] Xia Y Mao J Chen Y Wang D Cao L Yao S Clinical outcomes in children with Henoch-Schönlein purpura nephritis grade IIIa or IIIb. Pediatr Nephrol. (2011) 26(7):1083–8. 10.1007/s00467-011-1834-9 21387156

[24] Farzamikia N Baradaran B Mostafavi S Ahmadian E Hosseiniyan Khatibi SM Zununi Vahed S Podocyte-derived microparticles in IgA nephropathy. Biomed Pharmacother. (2021) 141:111891. 10.1016/j.biopha.2021.111891 34237594

[25] Pillebout E. IgA vasculitis and IgA nephropathy: same disease? J Clin Med. (2021) 10(11):2310. 10.3390/jcm10112310 34070665 PMC8197792

